# USP47 Promotes Tumorigenesis by Negative Regulation of p53 through Deubiquitinating Ribosomal Protein S2

**DOI:** 10.3390/cancers12051137

**Published:** 2020-05-01

**Authors:** Jinhong Cho, Jinyoung Park, Sang Chul Shin, Mihue Jang, Jae-Hong Kim, Eunice EunKyeong Kim, Eun Joo Song

**Affiliations:** 1 Biomedical Research Institute, Korea Institute of Science and Technology, Hwarangno 14-gil 5, Seongbuk-gu, Seoul 02792, Korea; wlsghd1116@gmail.com (J.C.); scshin84@kist.re.kr (S.C.S.); mihue@kist.re.kr (M.J.); 2 Department of Biotechnology, College of Life Sciences and Biotechnology, Korea University, 5-1 Anam-dong, Sungbuk-gu, Seoul 02841, Korea; jhongkim@korea.ac.kr; 3 Molecular Recognition Research Center, Korea Institute of Science and Technology, Hwarangno 14-gil 5, Seongbuk-gu, Seoul 02792, Korea; jypark@kist.re.kr; 4 Graduate School of Pharmaceutical Sciences, College of Pharmacy, Ewha Womans University, Seoul 03760, Korea

**Keywords:** USP47, RPS2, ribosomal stress, MDM2, p53

## Abstract

p53 is activated in response to cellular stresses such as DNA damage, oxidative stress, and especially ribosomal stress. Although the regulations of p53 by E3 ligase and deubiquitinating enzymes (DUBs) have been described, the cellular roles of DUB associated with ribosomal stress have not been well studied. In this study, we report that Ubiquitin Specific Protease 47 (USP47) functions as an important regulator of p53. We show that ubiquitinated ribosomal protein S2 (RPS2) by Mouse double minute 2 homolog (MDM2) is deubiquitinated by USP47. USP47 inhibits the interaction between RPS2 and MDM2 thereby alleviating RPS2-mediated suppression of MDM2 under normal conditions. However, dissociation of USP47 leads to RPS2 binding to MDM2, which is required for the suppression of MDM2, consequently inducing up-regulation of the p53 level under ribosomal stress. Finally, we show that depletion of USP47 induces p53 and therefore inhibits cell proliferation, colony formation, and tumor progression in cancer cell lines and a mouse xenograft model. These findings suggest that USP47 could be a potential therapeutic target for cancer.

## 1. Introduction

The tumor suppressor p53 acts as a key regulator of apoptosis and cell cycle control. Mutation or dysregulation of p53 can promote tumorigenesis and is highly correlated with poor prognosis of cancer. Various cellular stresses such as oxidative stress, DNA damage, and mitotic catastrophe trigger activation of p53, which in turn functions as a transcription factor, inducing its target genes related to apoptosis, cell growth arrest, and senescence [1,2]. The expression and activity of p53 is mainly regulated by MDM2, an E3 ligase [3]. Under normal conditions, the level of p53 is maintained at low levels by MDM2-mediated ubiquitination and degradation through the ubiquitin-proteasome system. Under stress conditions, however, the level of p53 is increased via the inhibition of MDM2 [4]. 

Recently, with an increased understanding of deubiquitination, several mechanisms of regulation of p53 and MDM2 by deubiquitinating enzymes (DUBs) have been described, with some DUBs regulating the MDM2–p53 axis through the direct deubiquitination of p53 or MDM2. They include Ubiquitin Specific Protease 7 (USP7) [5,6], USP10 [7], USP24 [8], USP42 [9], USP2a [10], and few others. In the case of USP7, which has higher similarity with USP47 [11], it was first identified as a DUB of p53 [5], but later it was found to be a more effective DUB for MDM2 [6]. Interestingly, USP10 has been shown to regulate the localization as well as the stabilization of p53 in response to genotoxic stress by the direct deubiquitination of p53 [7]. On the other hand, some DUBs are known to modulate p53 activity through the deubiquitination of p53 regulators other than MDM2. For example, USP4 regulates p53 levels by stabilizing ARF-BP1, a process crucial for ARF-mediated p53 ubiquitination [12], and USP22 suppresses p53 activation by deubiquitinating the histone deacetylase SIRT1, thereby antagonizing p53 transcriptional activity [13]. In another mechanism, OTUB1 suppresses the ubiquitin-conjugating activity of an E2 enzyme and subsequently regulates p53 levels [14]. 

Activation of p53 is also known to be induced by ribosomal stress, i.e., perturbation of ribosome biogenesis. Many studies have reported correlations between MDM2 regulation and ribosomal proteins [15]. In response to the stress, ribosomal proteins inhibit MDM2 by protein–protein interaction, resulting in p53 activation and progression of the p53 downstream pathway. For example, ribosomal proteins RPL11 and RPL5 have been shown to regulate the activation of p53 through the inhibition of MDM2 during ribosomal stress [16], while RPS2, RPS7, and RPS27a are substrates of MDM2 E3 ligase activity, and ubiquitinated ribosomal proteins inhibit MDM2 to induce p53 as a response pathway for ribosomal stress [17,18,19]. Especially, recent studies revealed that MDM2-dependent ubiquitination of RPS2 is K63-linked poly-ubiquitination, which means it can participate in the cellular signaling pathway, thereby inhibiting MDM2-dependent ubiquitination of p53 [19]. Therefore, the ribosomal protein–MDM2–p53 pathway can monitor the molecular integrity of ribosomal biogenesis. Nevertheless, the effect of deubiquitination associated with ribosomal stress has not been yet elucidated.

In this study, we identified RPS2 as a USP47 interacting protein. USP47 deubiquitinates RPS2 and alleviates RPS2-mediated inhibition of MDM2. In addition, ribosomal stress decreases the interaction between RPS2 and USP47, and dissociated RPS2 can then inhibit MDM2 and subsequently lead to p53 stabilization. By this mechanism, depletion of USP47 inhibits cancer cell growth and colony formation in a p53-dependent manner and it was shown to attenuate tumorigenesis in a xenograft mouse model. Therefore, these findings suggest that USP47 functions as a new regulator of the MDM2-p53 axis.

## 2. Results

### 2.1. USP47 Interacts with RPS2

**Figure 1:**
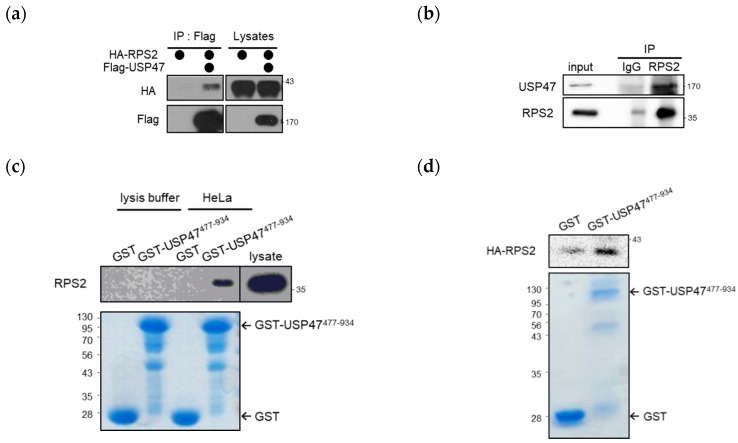
Ubiquitin Specific Protease 47 (USP47) interacts with ribosomal protein S2 (RPS2). (**a**) HEK293T cells were co-transfected with Flag-USP47 and HA-RPS2, and the cell lysates were immunoprecipitated with anti-Flag M2 agarose and detected with the indicated antibodies. (**b**) HEK293T cell lysates were immunoprecipitated with control IgG or anti-RPS2 antibodies, then the immunoprecipitates were detected with the indicated antibodies. (**c**) GST or GST-USP47^477–934^ was immobilized on glutathione beads and incubated with HeLa cell lysates. Bound proteins were separated in SDS-PAGE and immunoblotted with anti-RPS2 antibodies. (**d**) GST or GST-USP47^477–934^ was immobilized on glutathione beads and incubated with in vitro transcribed and translated HA-RPS2. Bound proteins were separated by SDS-PAGE and detected by Western blotting and Coomassie staining. The uncropped blots and molecular weight markers are shown in Figure S7.

In recent years, the USP family has emerged as an important target for anti-cancer drugs, and numerous studies of USP7 inhibitors are ongoing [20,21,22,23]. However, the cellular mechanisms of USP47 have not been well studied compared with other USPs, although USP47 has high similarity with USP7. Therefore, we focused on the cellular role of USP47. To determine the cellular function of USP47, we determined which proteins interact with USP47. First, USP47-interacting proteins were identified and isolated by mass spectrometry and RPS2, and a component of ribosomes was identified as an interacting protein. To verify the interaction of USP47 and RPS2, we performed immunoprecipitation with anti-Flag agarose after overexpressing HA-RPS2 and Flag-USP47. As shown in Figure 1a, USP47 interacted with RPS2 in cells. Moreover, ectopically overexpressed Flag-USP47 interacted with endogenous RPS2 (Figure S1). Consistent with these results, we also found that endogenous RPS2 interacted with endogenous USP47 in cells (Figure 1b). Next, we examined the direct interaction between USP47 and RPS2 by a Glutathione S-transferase (GST) pull-down assay using the GST-USP47^477–934^ protein that includes the ubiquitin-like (UBL) domain, as the UBL domain is known to mediate protein–protein interaction [24]. RPS2 in the cell lysate (Figure 1c) and RPS2 translated by in vitro transcription/translation (IVT/T) (Figure 1d) both bound to GST-USP47^477-934^. These results indicate that USP47 directly interacts with RPS2.

### 2.2. USP47 Regulates p53 in an MDM2-Dependent Manner

**Figure 2:**
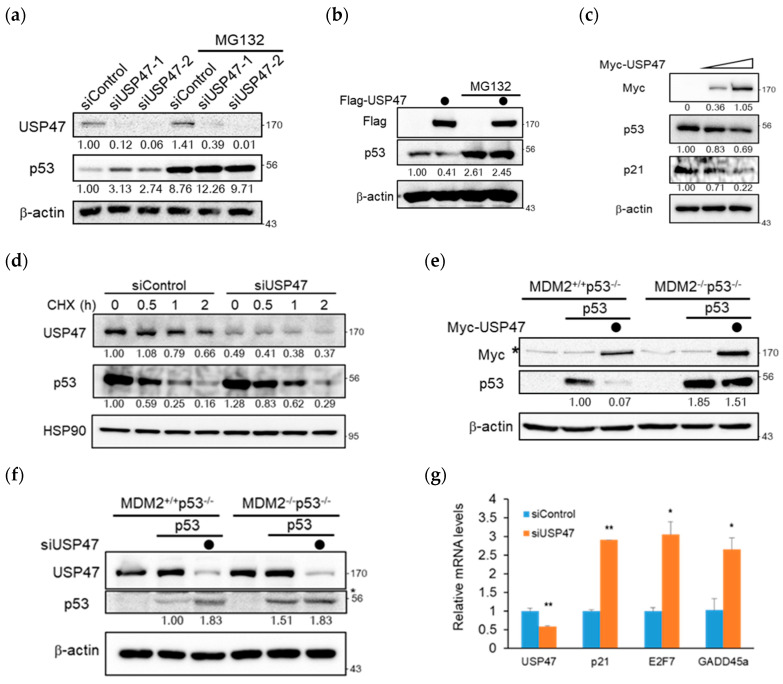
USP47 regulates p53 in an MDM2-dependent manner. (**a**) HeLa cells transfected with siRNA against control or USP47 were treated with either DMSO (control) or MG132 (10 μM) for 4 h. Cell lysates were immunoblotted with the indicated antibodies. β-actin was used as a loading control. The band intensity was measured by Image J and then normalized by β-actin. (**b**) U2OS cells overexpressed with Flag vector or Flag-USP47 were treated with either DMSO (control) or MG132 (10 μM) for 4 h. Cell lysates were immunoblotted with the indicated antibodies. (**c**) U2OS cells were overexpressed with increasing amounts of Myc-USP47 (0, 1, and 2 μg). After 24 h, cell lysates were immunoblotted with the indicated antibodies. (**d**) U2OS cells transfected with siRNA against control or USP47 were treated with 100 μg/mL cycloheximide and harvested at the indicated times. Cell lysates were immunoblotted with the indicated antibodies. HSP90 was used as a loading control. The band intensity was normalized by HSP90. (**e**) MDM2^+/+^p53^−/−^ and MDM2^−/−^p53^−/−^ Mouse Embryonic Fibroblast (MEF) cell lines were co-overexpressed with p53, Myc-USP47, and then cell lysates were immunoblotted with the indicated antibodies. The non-specific band is denoted by an asterisk. (**f**) MDM2^+/+^p53^−/−^ and MDM2^−/−^p53^−/−^ Mouse Embryonic Fibroblast (MEF) cell lines were overexpressed with p53 for 24 h and then transfected with siRNA against control or USP47 for 48 h. Cell lysates were immunoblotted with the indicated antibodies. The non-specific band is denoted by an asterisk. (**g**) The real-time PCR analysis of p53 downstream genes in A549 cells transfected with siRNA against control or USP47 for 48 h. Relative expression values are normalized against β-actin RNA levels, and graphs are shown as fold induction over control siRNA transfected cells. Data from three independent experiments represented by mean ± SD (* *p* < 0.05, ** *p* < 0.01, *t*-test). The uncropped blots and molecular weight markers are shown in Figure S7.

Since the previous study already showed that RPS2 increased p53 stability [19], we examined whether USP47 affected the protein level of p53. As shown in Figure 2a, the depletion of USP47 increased the p53 level but not in the presence of MG132. When tested using the colorectal cancer cell line, HCT116, the p53 level was also increased by USP47 depletion (Figure S2a). In contrast, the overexpression of Flag-USP47 decreased the p53 level, and it was also rescued by treatment with MG132 (Figure 2b). To clarify the effect of USP47 on the p53 level, we ectopically overexpressed the increasing amounts of Myc-USP47. The result showed that the p53 level decreased slightly in a dose-dependent manner, and p21, a p53 downstream protein, showed a more decreasing pattern than p53 (Figure 2c). To examine whether USP47 affected the stability of p53, we performed a degradation assay after the depletion of USP47 (Figure 2d). The degradation of p53 was delayed when USP47 was depleted, which is shown more clearly in the graph (Figure S2b). Since the regulation of p53 by USP47 was proteasome-dependent (Figure 2a,b), we hypothesized that p53 regulation by USP47 could be dependent on MDM2. To test this, p53 was transiently overexpressed both with and without USP47, and its level was compared in Mouse Embryonic Fibroblast (MEF) cell lines lacking either Mdm2 and p53 genes or p53 gene, e.g., MDM2^+/+^p53^−/−^ and MDM2^−/−^p53^−/−^. Interestingly, p53 decrease by USP47 overexpression and p53 increase by USP47 knockdown were only shown in the MDM2^+/+^p53^−/−^ MEF cell line, while there were no significant p53 level changes in the MDM2^−/−^p53^−/−^ MEF cell line (Figure 2e,f). Since USP47 regulates p53 in an MDM2-dependent manner, it can also regulate the transcriptional activity of p53. To elucidate the USP47 effect on p53 activity, we examined real-time PCR to compare the gene expression level of p53-targeted downstream genes under USP47 knockdown. As expected, USP47 knockdown upregulated the transcriptional level of p53 downstream genes, such as p21, E2F7, and GADD45α (Figure 2g). Therefore, we conclude that USP47 inhibits p53 accumulation and its transcriptional activity in an MDM2-dependent manner. 

### 2.3. USP47 Deubiquitinates RPS2 and Regulates the Interaction between MDM2 and RPS2

**Figure 3:**
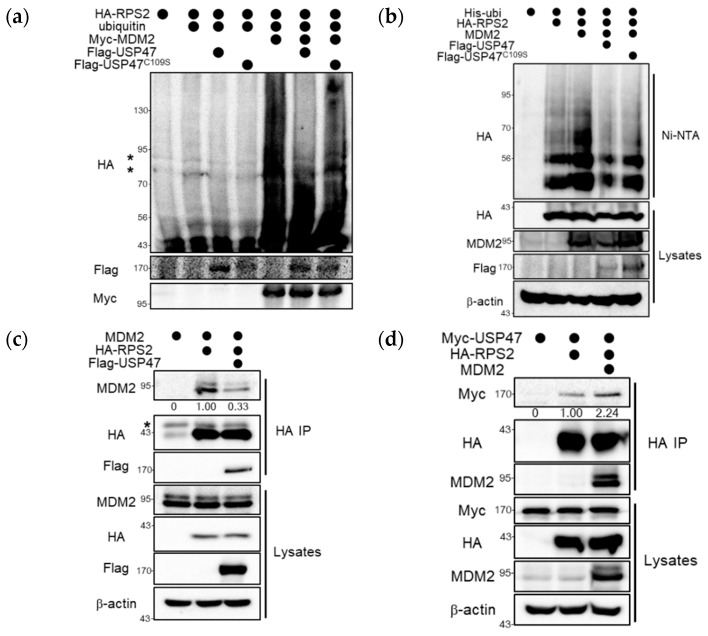
USP47 deubiquitinates RPS2 and regulates the interaction between RPS2 and MDM2. (**a**) HeLa cells were transfected with ubiquitin, HA-RPS2, Myc-MDM2, Flag-USP47, or Flag-USP47^C109S^ and then treated with MG132 (10 μM) for 4 h. Cells were harvested and immunoblotted with the indicated antibodies. The non-specific band is denoted by an asterisk. (**b**) HEK293T cells were co-overexpressed with His-ubiquitin, HA-RPS2, MDM2, Flag-USP47, or Flag-USP47^C109S^ and then treated with MG132 (10 μM) for 4 h. Ubiquitin conjugates were purified on Ni-NTA agarose under denaturing conditions, and ubiquitinated RPS2 was detected with the anti-HA antibody. (**c**) HEK293T cells were overexpressed with MDM2, HA-RPS2, and Flag-USP47, and then immunoprecipitation was performed with anti-HA agarose. Bound proteins were immunoblotted with the indicated antibodies. The non-specific band is denoted by an asterisk. (**d**) HEK293T cells were overexpressed with Myc-USP47, HA-RPS2, and MDM2, and then immunoprecipitation was performed with anti-HA agarose. Bound proteins were immunoblotted with the indicated antibodies. The uncropped blots and molecular weight markers are shown in Figure S7.

To elucidate the functional mechanism of USP47, we examined the effect of USP47 on the ubiquitinated RPS2 by MDM2. When RPS2 was co-expressed with MDM2 and ubiquitin, high-molecular-weight forms of RPS2 were detected consistent with a previous report (Figure 3a) [19], and these forms were significantly decreased in the presence of USP47 but not by the catalytically inactive USP47^C109S^. They represented a covalent modification of RPS2 with ubiquitin, as confirmed through denaturing Ni-NTA pull-down assays. The ubiquitinated RPS2 by MDM2 was deubiquitinated by USP47 but not by USP47^C109S^ (Figure 3b). Therefore, we conclude that RPS2 is a substrate of USP47. 

Recent reports show that there are upstream regulators of ribosomal proteins involved in the MDM2–p53 pathway, and these upstream regulators such as Glutamate Rich WD Repeat Containing 1 (GRWD1), PICT1 (also known as GLTSCR2), and Spindlin 1 (SPIN1) mainly have an effect on the interaction between ribosomal proteins and MDM2, which is important for p53 activation [25,26,27]. So, we examined whether USP47 had an effect on the interaction between RPS2 and MDM2. The results showed that RPS2 interacted with MDM2, but this interaction was decreased by overexpression of USP47 (Figure 3c). On the contrary, the interaction between USP47 and RPS2 was not decreased by the addition of MDM2 (Figure 3d and Figure S3). These results suggest that the presence of USP47 regulates the binding of RPS2 to MDM2, which is required for the inhibition of MDM2. 

### 2.4. USP47 Alleviates RPS2-mediated Inhibition of MDM2 by Regulation of Ubiquitination Status of RPS2

**Figure 4:**
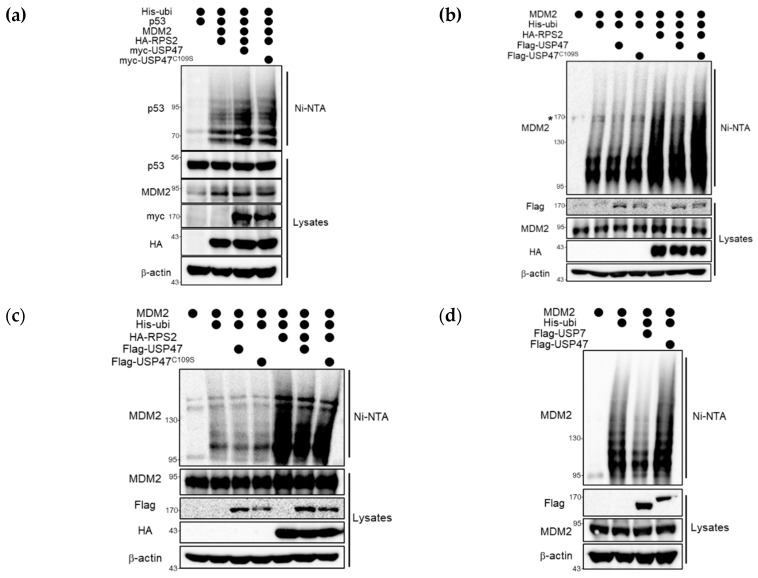
USP47 regulates the ubiquitination of p53 and MDM2. (**a**) HEK293T cells were co-overexpressed with His-ubiquitin, p53, MDM2, HA-RPS2, myc-USP47, or myc-USP47^C109S^ for 24 h and then treated with MG132 (10 μM) for 4 h. Ubiquitin conjugates were purified on NiNTA-agarose under denaturing conditions, and ubiquitinated p53 was detected with anti-p53 antibody. (**b**) U2OS cells co-overexpressing His-ubiquitin, HA-RPS2, MDM2, Flag-USP47, or Flag-USP47^C109S^ as indicated were treated with MG132 (10 μM) for 4 h. Ubiquitin conjugates were purified on Ni-NTA agarose under denaturing conditions, and ubiquitinated MDM2 was detected with the anti-MDM2 antibody. The non-specific band is denoted by an asterisk. (**c**) H1299 cells were co-overexpressed with MDM2, His-ubiquitin, HA-RPS2, Flag-USP47, or Flag-USP47^C109S^ and then treated with 10 μM MG132 for 4 h. Ubiquitin conjugates were purified on Ni-NTA agarose under denaturing conditions, and ubiquitinated MDM2 was detected by anti-MDM2 antibodies. (**d**) HEK293T cells were overexpressed with MDM2, His-ubiquitin, and Flag-USP7 or Flag-USP47 and then treated with 10 μM MG132 for 4 h. Ubiquitin conjugates were purified on Ni-NTA agarose under denaturing conditions, and ubiquitinated MDM2 was detected by anti-MDM2 antibodies. The uncropped blots and molecular weight markers are shown in Figure S7.

In the above, we confirmed that USP47 affects regulating MDM2-dependent p53 protein level (Figure 2e,f), RPS2 ubiquitination (Figure 3a,b), and RPS2-MDM2 interaction (Figure 3c). To demonstrate more details of their mechanisms, we examined whether USP47 affected RPS2-mediated inhibition of MDM2. The ubiquitination of p53 or MDM2 was examined, and the results showed that p53 ubiquitination by MDM2 was increased by USP47 but not by USP47^C109S^ in the presence of RPS2 (Figure 4a). 

Next, we investigated whether USP47 had an effect on MDM2 ubiquitination as well as p53 ubiquitination. As shown in Figure 4b, there was no effect on the ubiquitination of MDM2 when USP47 alone was overexpressed in the absence of RPS2. However, co-overexpression of RPS2, which is known to inhibit MDM2 and subsequently induces p53 activation [19], increased the ubiquitination of MDM2, and this effect was restored by the co-expression of USP47 but not by USP47^C109S^. Furthermore, increased MDM2 ubiquitination by RPS2 and its restoration by USP47 were also confirmed in the HEK293T cell line as well (Figure S4). Notably, in the MDM2–p53 pathway, there is positive feedback from p53 to MDM2. To exclude such a positive feedback effect on MDM2 ubiquitination from the increased p53, we performed the same experiment with a p53-deficient H1299 cell line in Figure 4c. Consistent with the results in Figure 4b, RPS2 increased MDM2 ubiquitination, and this effect was restored in the presence of USP47 in H1299 cells. These results suggest that USP47 regulates RPS2-mediated MDM2 inhibition through deubiquitinating RPS2, consequently regulating the MDM2–p53 pathway. However, this finding raises a question: does USP47 directly deubiquitinate MDM2? To answer this, we performed denaturing Ni-NTA pull-down assays against MDM2 to assess the effect of USP7 and USP47 (Figure 4d). Contrary to USP7, USP47 did not have any effect on ubiquitinated MDM2 without RPS2 [17]. Based on these results, we conclude that USP47 leads to a decrease in ubiquitinated MDM2 as a result of RPS2-mediated regulation, not direct deubiquitination. Taken together, these results indicate that USP47 regulates the MDM2–p53 pathway through the deubiquitination of RPS2.

### 2.5. Ribosomal Stress Inhibits the Interaction between USP47 and RPS2 and thus Increases p53

**Figure 5:**
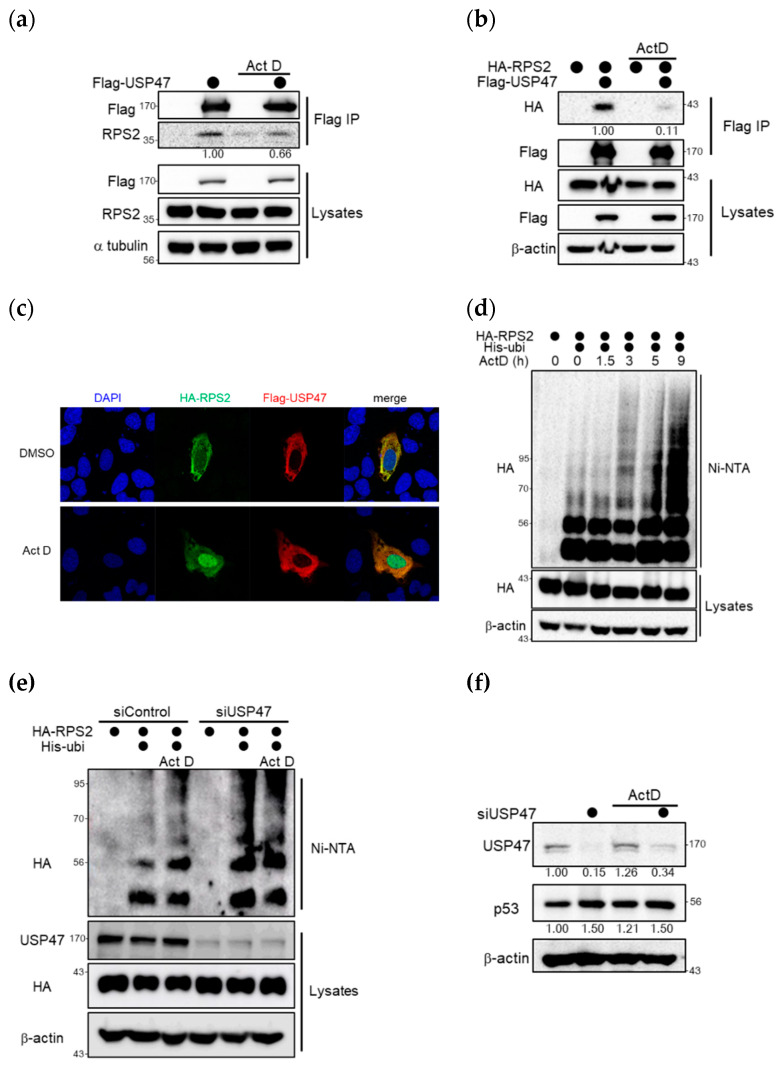
Ribosomal stress leads to RPS2 ubiquitination for MDM2 regulation to induce p53 by dissociation of USP47 and RPS2. (**a**) U2OS cells co-overexpressing either Flag vector or Flag-USP47 were treated with DMSO or 5 nM actinomycin D for 4 h, and immunoprecipitation was performed with anti-Flag agarose. Bound proteins were immunoblotted with the indicated antibodies. α-tubulin was used as a loading control. (**b**) HEK293T cells were co-overexpressed with HA-RPS2, Flag-USP47, and then treated with DMSO or 5 nM actinomycin D for 4 h. Then, cell lysates were immunoprecipitated with anti-Flag M2 agarose and detected with the indicated antibodies. (**c**) U2OS cells were co-overexpressed with Flag-USP47 and HA-RPS2 and then treated with DMSO or 5 nM actinomycin D for 4 h. Cells were immunostained with anti-HA antibody followed by Alexa Fluor 488 anti-mouse IgG and Flag antibodies followed by Alexa Fluor 546 anti-rabbit IgG. Representative immunofluorescence images were captured by confocal microscopy. (**d**) HEK293T cells were overexpressed with HA-RPS2 and His-ubiquitin and then treated with 10 μM MG132 for 4 h and 5 nM actinomycin D for indicated times. Ubiquitin conjugates were purified on Ni-NTA-agarose under denaturing conditions, and ubiquitinated RPS2 was detected by anti-HA antibodies. (**e**) U2OS cells were co-transfected with His-ubiquitin, HA-RPS2, and either siControl or siUSP47 and then treated with 10 μM MG132 and either DMSO or 5 nM actinomycin D for 4 h. Ubiquitin conjugates were purified on Ni-NTA agarose under denaturing conditions, and ubiquitinated RPS2 was detected by anti-HA antibodies. (**f**) U2OS cells transfected with siRNA against control or USP47 were treated with either DMSO or 5 nM actinomycin D for 4 h. Cell lysates were immunoblotted with the indicated antibodies. The uncropped blots and molecular weight markers are shown in Figure S7.

Since the upregulation of p53 by ribosomal proteins mainly occurred under ribosomal stress, we compared the interaction between USP47 and RPS2 under normal or ribosomal stress. As shown in Figure 5a, overexpressed USP47 interacted with endogenous RPS2, but this interaction was decreased under treatment with actinomycin D, which is a ribosomal stress inducer. Moreover, the dissociation of the interaction was more dramatic between overexpressed RPS2 and USP47 (Figure 5b). Next, to analyze subcellular localization of USP47 and RPS2 under normal and ribosomal stress conditions, immunocytochemistry experiments were performed. As shown in Figure 5c, USP47 was localized in the cytosol, and RPS2 was dispersed throughout the whole cell in normal conditions. During ribosomal stress, RPS2 was accumulated in the nucleus and USP47 remained localized in the cytosol. Localization data also showed that interaction between USP47 and RPS2 dissociated during ribosomal stress, as shown in Figure 5a,b. MDM2 is localized in the nucleus during both normal and ribosomal stress conditions [28,29]. Therefore, RPS2 can effectively regulate MDM2 effectively during ribosomal stress. Ubiquitination of both overexpressed and endogenous RPS2 was accumulated with actinomycin D treatment in a time-dependent manner, as shown in Figure 5d and Figure S5. The accumulation of ubiquitinated RPS2 may lead to MDM2 inhibition and increase p53. 

Next, we examined RPS2 ubiquitination in USP47-depleted cells (Figure 5e). The ubiquitination of RPS2 in USP47-depleted cells was increased both under normal and with ribosomal stress conditions. This result indicates that RPS2 could not be deubiquitinated by USP47 with depleted USP47. Accordingly, there is no significant difference in the ubiquitination of RPS2 between normal and ribosomal stress conditions in USP47-depleted cells. Therefore, the ubiquitination of RPS2 was increased by either USP47 depletion or ribosomal stress. 

To investigate the further effect of ribosomal stress, we checked the protein level of p53 in the depletion of USP47. Ribosomal stress upregulated p53 but did not show any dramatic effect in increasing p53 since MDM2 could already be inhibited by RPS2 in the depletion of USP47 (Figure 5f). Taken together, these results suggest that USP47 inhibits the interaction between RPS2 and MDM2, thereby decreasing RPS2-mediated inhibition of MDM2 under normal conditions ( Figure 2; Figure 3). However, dissociation of RPS2 from USP47 led to the accumulation of RPS2 ubiquitination, which is required for the inhibition of ubiquitin ligase activity of MDM2, consequently inducing up-regulation of the p53 level under ribosomal stress.

### 2.6. Suppression of USP47 Inhibits Cancer Cell Growth

**Figure 6:**
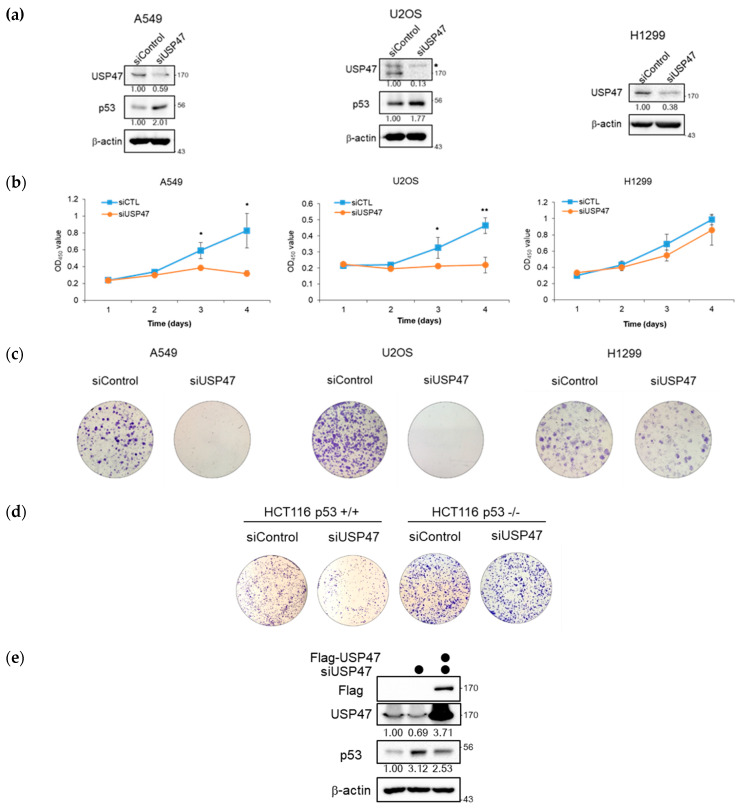

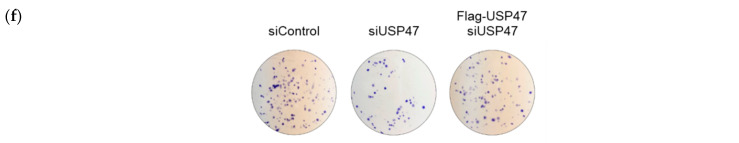
Suppression of USP47 inhibits cancer growth in vitro. (**a–c**) A549, U2OS, and H1299 cells were transfected with siRNA against control or USP47. (**a**) Then, cell lysates were immunoblotted with the indicated antibodies. (**b**) Cell viability was measured using EX-Cytox assay kit. Data from three independent experiments represented by mean ± SD (* *p* < 0.05, ** *p* < 0.005, *t*-test). (**c**) Colony formation assay was performed by re-seeding cells after 48 h transfection and then fixed and stained with crystal violet after 10 days. (**d**) Colony formation assay was performed in HCT116 p53^+/+^ and p53^−/−^ cell lines transfected with negative control or USP47 siRNA. Cells were re-seeded after 48 h and then fixed and stained with crystal violet after 10 days. (**e**,**f**) A549 cells were overexpressed with Flag-USP47 for 24 h and then transfected with siRNA against control or USP47 for 48 h. (**e**) Cell lysates were immunoblotted with the indicated antibodies. (**f**) Colony formation assay was performed by re-seeding cells after 48 h transfection and then fixed and stained with crystal violet after 10 days. The uncropped blots and molecular weight markers are shown in Figure S7.

It has been shown that p53 regulates many of the cellular pathways such as inhibition of cell cycle progression, cell proliferation, and induction of apoptosis. Since USP47 is overexpressed in colon cancer [30] and involved in the regulation of p53 signaling, we questioned whether USP47 regulates cancer cell viability p53 dependently. For this, we used both p53-positive and p53-deficient cells, namely A549 and U2OS cells, and H1299 cells, respectively. After the transfection of control or USP47 siRNAs, we performed MTT cell proliferation assays and colony formation assays, and the results are shown in Figure 6a–c. When USP47 was depleted, cell proliferation was totally blocked and colonies were not formed in A549 and U2OS cell lines. However, in H1299, the p53 null cell line, depletion of USP47 had no significant effect on cell proliferation or few reduction effects on colony formation, although USP47 was depleted very well (Figure 6a–c). 

To confirm whether the effect of USP47 on cell proliferation was p53 dependent or not, the effect of USP47 depletion was compared using both HCT116 p53^+/+^ and HCT116 p53^−/−^ cell lines. USP47 depletion in HCT116 p53^+/+^ wild type cell line inhibited colony formation (Figure 6d), but there was no difference by USP47 depletion in the p53-ablated HCT116 p53^−/−^ cell line. These results showed that USP47 regulates cancer cell growth in a p53-dependent manner. We then performed the rescue experiment. The depletion of USP47 increased p53 protein level and decreased colony formation, consistent with Figure 6a,c, and increased p53 protein level was decreased by the overexpression of USP47 (Figure 6e,f). Together, these results indicate that the cancer cell phenotype by USP47 depletion was not caused by off-target effects.

**Figure 7:**
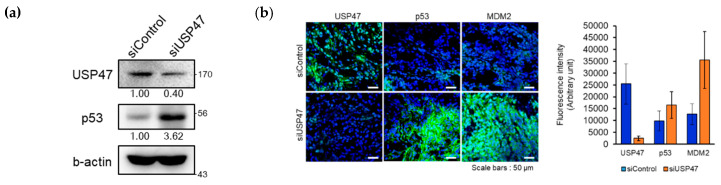

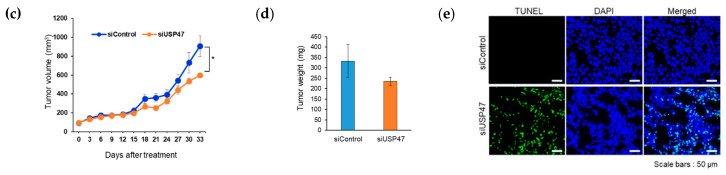
Suppression of USP47 inhibits cancer growth in vivo. (**a**) Western blot was conducted with tumor samples to evaluate the levels of USP47, p53, detected by indicated antibodies. (**b**) Immunofluorescence staining was conducted in dissected tumors to evaluate the levels of USP47, p53, MDM2 and detected by indicated antibodies. Immunofluorescence images were captured by confocal microscopy and quantified by ImageJ. Blue colors represent DAPI. Scale bars indicate 50 µm. (**c–d**) The tumor growth curves after initial treatment were shown; 2 mg/kg of siControl and siUSP47 were intratumorally administered into A549 xenografts every 3 days for 2 weeks. Tumor volumes were measured every 3 days after initial treatment. * *p* < 0.5 (*N* =4). Tumor weights were measured. (**e**) TUNEL assay was performed in dissected tumors to detect apoptotic cells using an *in situ* cell death detection kit. Blue colors represent DAPI. Scale bars indicate 50 µm. The uncropped blots and molecular weight markers are shown in Figure S7.

Next, to determine the role of USP47 in regulating cancer growth in vivo, scrambled siRNA (siControl) and USP47 siRNA were locally administered into A549 xenografts. We examined the expression of the two on tumor sites using Western blotting and immunostaining assay to evaluate the behavior of p53 under the depletion of USP47. Consistent with in vitro data, p53 was highly accumulated in a tumor administered with siUSP47 in the Western blotting (Figure 7a) and immunostaining assay (Figure 7b and Figure S6a). Moreover, MDM2 as well as p53 were accumulated in a tumor administered with siUSP47 (Figure 7b and Figure S6a). MDM2 stabilization in vivo was eventually followed by p53 positive feedback, caused by long-term p53 induction because MDM2 is also a target gene of p53 transcriptional activity. Compared with xenografts receiving the scrambled siRNA, xenografts receiving the siUSP47 exhibited a decrease in tumor volume and weight (Figure 7c,d and Figure S6b). The treatment of siUSP47 suppressed tumor growth by 40%. Additionally, significantly induced apoptotic cells were observed when USP47 was inhibited, as demonstrated by TUNEL assay (Figure 7e). Thus, the findings here indicate that USP47 can promote tumor proliferation by regulating levels of p53.

## 3. Discussion

**Figure 8:**
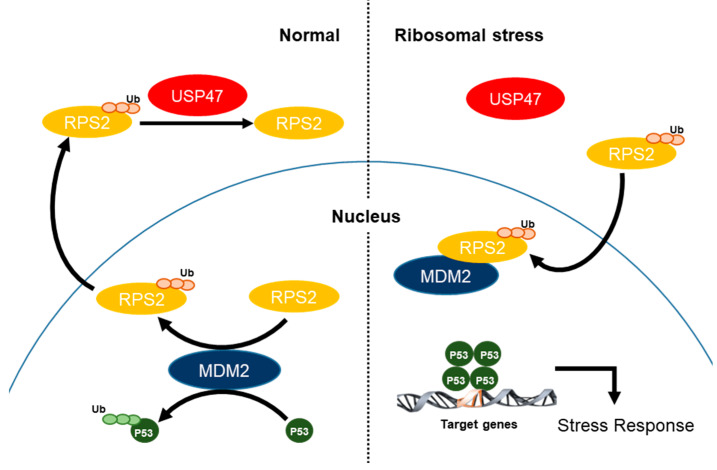
A schematic overview. Under normal conditions, USP47 deubiquitinates RPS2, and thus MDM2 inhibits p53 to maintain p53 protein levels. Under ribosomal stress, USP47 dissociates from RPS2, and thus ubiquitination of RPS2 is accumulated thereby inhibiting MDM2 to induce p53 protein levels for turning on the stress response signal.

Thus far, very little is known about USP47 in cancer, even though USP47 has high sequence similarity with USP7 of whose inhibitors are now actively developed as an anticancer drug [20,21,22]. In fact, to the best of our knowledge, there are only a few reports that show a direct relationship, that is USP47 is overexpressed in colorectal cancer and depletion of USP47 inhibits colon cancer progression [30] and induces deficiency in base excision repair, leading to accumulation of DNA strand breaks [31]. Moreover, USP47 is involved in cell survival [32] and cell viability [31]. To further understand the cellular role of USP47, we investigated its interacting proteins and related mechanism in detail. In this study, we found RPS2 is a substrate of USP47 DUB activity, and USP47 counteracts MDM2 to RPS2 ubiquitination, which in turn can regulate p53. Through the deubiquitination of RPS2, USP47 regulates the interaction between RPS2 and MDM2, and USP47 functions as an important regulator of the MDM2–p53 axis in ribosomal stress. Finally, we demonstrated that USP47 suppressed tumors in a p53-dependent manner by this USP47–RPS2–MDM2–p53 mechanism (Figure 8). 

Several studies have reported on the molecular mechanism of how ribosomal proteins inhibit MDM2. For instance, the inhibition of the interaction between MDM2 and p53 [33,34], the upregulation of p53 mRNA translation [35], the prevention of the co-ribosomal export of p53 and MDM2 [36], or the regulation by 5S ribonucleoprotein particle (RNP) have all been suggested as regulatory mechanisms by ribosomal proteins [37]. However, it is not fully understood why MDM2 activity is maintained against regulation by ribosomal proteins under normal conditions and is inhibited by ribosomal proteins only after ribosomal stress. It has been reported that PICT1 functions as an anchor of RPL11 in the nucleolus, thus preventing RPL11 from inhibiting MDM2 in the nucleoplasm and releasing RPL11 into the nucleoplasm in response to ribosomal stress [26]. GRWD1 interacts with RPL11 and competitively inhibits the RPL11–MDM2 interaction, which is required for the RPL11-mediated suppression of MDM2 activity [25]. In our study, we showed that USP47 is a determining factor for the regulation of the MDM2–p53 pathway by RPS2 in ribosomal stress; that is, USP47 deubiquitinates RPS2 and competitively inhibits the binding between RPS2 and MDM2, thereby maintaining the E3 ligase activity of MDM2 to inactivate p53 under normal cell conditions. In contrast, under ribosomal stress conditions, USP47 is released, and the accumulation of ubiquitinated RPS2 inhibits MDM2 by inducing ubiquitination of MDM2; this, in turn, activates p53 in response to ribosomal stress. As such, USP47 functions as a delicate regulator when the cell needs to stabilize p53 in response to cellular stress such as ribosomal stress. This finding explains why USP47 is needed in the cell to maintain the homeostasis of p53 and also suggests that perturbations of the expression level of USP47 may be the cause of cancer. 

USP7 has already been widely studied as an effective target for anti-tumor therapies, in that USP7 stabilizes MDM2 and thereby downregulates p53 [6,38]. Research on numerous USP7 inhibitors is ongoing, and some inhibitors are undergoing clinical testing as anti-cancer drug candidates [39,40]. As we mentioned briefly above, USP47 has a higher similarity with USP7; thus, some USP7 inhibitors are dual inhibitors for USP7 and USP47 [41]. Therefore, USP47 may have strong potential as a p53 regulator, and we demonstrate this with our experimental data. We suggest that USP47 also can be a good target for the anti-cancer drug in that, like USP7, it also regulates p53, even by different mechanisms for p53 regulation. Whereas USP7 stabilizes MDM2 directly, it regulates MDM2 by indirect interaction: by deubiquitinating RPS2. Therefore, USP47 is a delicate regulator of p53; thus, an inhibitor for USP47 might be an important and effective anti-cancer drug. Furthermore, the differences between USP7 and USP47 based on their structures or mechanisms should be further studied.

## 4. Materials and Methods 

### 4.1. Cell Lines and Transfection

HEK293T cells, A549 cells, U2OS cells, HeLa cells, and H1299 cells were purchased from Korea Cell Line Bank (KCLB), and MDM2^−/−^p53^−/−^ and MDM2^+/+^p53^−/−^ Mouse Embryonic Fibroblast cell lines were kindly supplied by G. Lozano [42]. HeLa cells, HEK293T cells, and Mouse Embryonic Fibroblast MDM2^−/−^p53^−/−^ and MDM2^+/+^p53^−/−^ cell lines were grown in 10% fetal bovine serum, 100 units/mL penicillin and 100 μg/mL streptomycin, Dulbecco’s Modified Eagle’s Medium (DMEM). U2OS cells, A549 cells, and H1299 cell lines were grown in 10% fetal bovine serum, 100 units/mL penicillin and 100 μg/mL streptomycin, Roswell Park Memorial Institute 1640 (RPMI 1640). Cells were grown in 5% CO2 and 95% air at 37 °C. 

Either Effectene (Qiagen, Hilden, Germany) or Mirus LT-1 (Mirus, Madison, WI, USA) were used for DNA plasmid transfection following the manufacturer’s instructions in other cell lines. The calcium phosphate/DNA co-precipitation method [43] was used for DNA plasmid transfection in the HEK293T cell line. Lipofectamine2000 (Invitrogen, Carlsbad, CA, USA) was used for siRNA transfection according to the manufacturer’s instructions. 

The control siRNA sequence was 5′-CCUACGCCACCAAUUUCGU-3′. The USP47 siRNA sequences were #1 5′-GTGCAAAGGCCATGAATGA-3′ and #2 5′-TGAAAAGGGATGTGCAAAA-3′’ (Genolution, Seoul, Korea). 

### 4.2. Plasmids

The coding sequence for human USP47, RPS2, and ubiquitin were amplified by PCR and cloned into the pCS2 vector plasmid, which incorporates HA or Myc tag at the N terminus. pFlag-CMV^TM^-2 vector, pFlag-CMV^TM^-2-USP47, pFlag-CMV^TM^-2-USP47^C109S^ (catalytically inactive mutant), and pFlag-USP7 were kindly provided by Dr. CH Chung [44]. pCS2-Myc-MDM2, pCMV-MDM2, and pCMV-MDM2^C464A^ were kindly given by Dr. SH Baek [45]. Histidine-tagged ubiquitin and various mutants were prepared as previously described [46].

### 4.3. Antibodies and Reagents

The following antibodies were purchased from following companies and used for Western blotting and immunostaining. Antibodies for USP47 and RPS2 were from Bethyl Laboratories (Montgomery, TX, USA). Antibodies for HA, Myc, p53, and p21 were from Santa Cruz Biotechnology. Antibody for Flag was from Sigma Aldrich (St. Louis, MO, USA). Antibody for MDM2 was from Calbiochem (San Diego, CA, USA). Antibody for *β*-actin was from Ab Frontier. After Western blotting, the band intensity was measured by Image J and then was normalized by *β*-actin. The following drugs were purchased from following companies and were treated to cells for the following reasons. MG132 (A.G scientific, San Diego, CA, USA) was treated at a 10 μM concentration to inhibit proteasomal degradation. Actinomycin D (Merck, Kenilworth, NJ, USA) was treated at a 5 nM concentration to induce ribosomal stress. Cycloheximide was treated at a 100 μg/mL concentration to inhibit protein synthesis. 

### 4.4. Immunoprecipitation

Harvested cells were lysed with cell lysis buffer (pH 7.4, 150 mM NaCl, 50 mM Tris-Cl, 1 mM EDTA, 0.5% NP-40, 1 mM sodium orthovanadate, and protease inhibitor cocktail; Roche, Basel, Switzerland). BCA kit (Thermo Scientific, Waltham, MA, USA) was used for measuring protein concentrations. EZview^TM^ Red anti-Flag M2 affinity gel (Sigma Aldrich) or monoclonal anti-HA agarose conjugate (Sigma Aldrich) were incubated with 2 mg of cell lysates for 4 h at 4 °C and washed three times with lysis buffer. For endogenous immunoprecipitation, rabbit IgG (sc-2027, Santa Cruz Biotechnology, Dallas, TX, USA) or rabbit anti-RPS2 were incubated with 10 mg of HEK293T cell lysates for 4 h at 4 °C. Then, protein A agarose beads (Gendepot, Barker, TX, USA) were added into the conjugates of lysates–antibody and incubated for 12 h at 4 °C. The supernatant was removed carefully with a syringe after the last wash. For Flag-IP, the agarose was incubated with 3× Flag peptide (Sigma Aldrich) in elution buffer (15 μg of Flag peptide in 50 μL of lysis buffer) for 30 min; then, bound proteins were eluted to the supernatants from the agarose. The eluted supernatant was boiled with 2× SDS sample buffer at 95 °C. For HA-IP, the agarose was eluted and boiled with 2× SDS sample buffer at 95 °C. Eluted proteins were separated by SDS-PAGE and submitted to Western blotting. After Western blotting, the band intensity was measured by Image J and then was normalized by a tag that was used for IP.

### 4.5. Ni-NTA Pulldown Assay

Harvested cells were lysed with urea lysis buffer (pH 8.0, 8 M urea, 50 mM Tris, 300 mM NaCl, 50 mM Na_2_HPO_4_, 1 mM phenylmethylsulfonyl fluoride (PMSF), and 10 mM imidazole) by sonication. Ni-NTA agarose (Qiagen) was incubated with cell lysates in a rotator for 4 h at 4 °C. The agarose was washed with urea lysis buffer (pH 8.0, 8 M urea, 50 mM Tris, 300 mM NaCl, 50 mM Na_2_HPO_4_, 1 mM PMSF, and 20 mM imidazole) five times. During the last wash step, the supernatants were carefully removed with a syringe. The agarose was eluted and boiled with 2× SDS sample buffer at 95 °C. Eluted proteins were separated by SDS-PAGE and submitted to Western blotting [47].

### 4.6. GST Pull-Down Assay

GST and GST-USP47^477–934^ were coupled to glutathione agarose (Sigma Aldrich) with coupling buffer (20 mM HEPES pH 7.5, 5 mM KCl, 3 mM MgCl_2_, 15 mM NaCl, 0.1% Tween 20, and 0.1% BSA) in a rotator for 3 h at 4 °C. Agarose was washed with SB buffer (20 mM HEPES pH 7.5, 5 mM KCl, and 3 mM MgCl_2_) three times and incubated with cell lysates in a rotator for 3 h at 4 °C. Agarose was washed three times with wash buffer 1 (150 mM NaCl and 0.1% Tween20 in SB buffer) and then washed twice with wash buffer 2 (150 mM NaCl in SB buffer). After the last wash step, supernatants were removed carefully by syringe. The agarose was eluted with 2× SDS sample buffer by boiling at 95 °C. Samples were separated by SDS-PAGE for Western blotting or silver staining (GE Healthcare, Chicago, IL, USA).

### 4.7. Quantitative Reverse Transcription-PCR Analysis

Total cellular RNA was extracted from cells using the RNeasy Mini Kit (Qiagen). cDNAs were synthesized by total RNA using ReverTra Ace qPCR RT Master Mix (Toyobo, Osaka, Japan) and analyzed with SYBR Green Real-time PCR Master Mix (Toyobo). The results were normalized to the expression of β-actin. Used primer sequences are given as follows. The USP47 primers were (F) 5′-ATGGAAGAATCCTGGCACTG-3′ and (R) 5′-CTGGAAGGGATCCAACTTCA-3′. The p21 primers were (F) 5′-CACCACTGGAGGGTGACTTC-3′ and (R) 5′-ATCTGTCATGCTGGTCTGCC-3′. The E2F7 primers were (F) 5′-GGAAAGGCAACAGCAAACTCT-3′ and (R) 5′-GAGAGCACCAAGAGTAGAAGA-3′. The GADD45α primers were (F) 5′-TGCGAGAACGACATCAACAT-3′ and (R) 5′-TCCCGGCAAAAACAAATAAG-3′. The β-actin primers were (F) 5′-CTCTTCCAGCCTTCCTTCCT-3′ and (R) 5′-AGCACTGTGTTGGCGTACAG-3′.

### 4.8. MTT Assay

Cell proliferation assay was performed using EX-Cytox (DoGen, #EZ3000, Seoul, Korea) according to the manufacturer’s instructions. Cells were seeded into 96-well plates at a density of 1000 to 3000 cells per well (A549: 1000 cells, U2OS: 3000 cells, H1299: 3000 cells/each well). After transfection with siControl or USP47 siRNA, EZ-Cytox reagents were added into cells and incubated for 30 min. Then, absorbance at 450 nm was measured using a Microplate Absorbance Reader (Bio-Rad, Hercules, CA, USA).

### 4.9. Colony Formation Assay

Cells were seeded in a 35 mm culture dish at different numbers depending on cell lines (A549: 5000 cells, U2OS: 40,000 cells, H1299: 5000 cells/each dish). The medium was changed every 3 days. After 10 days, cells were washed with PBS and fixed with 4% paraformaldehyde for 20 min, then they were washed with PBS and stained with crystal violet for 30 min and washed with PBS [48]. Cell dishes were scanned for the data.

### 4.10. A549 Tumor Xenografts

All animal experiments were performed in compliance with institutional guidelines as approved by the Institutional Animal Care and Use Committee of KIST (ethic code: KIST-2019-004). For tumorigenesis, 1 × 10^7^ of A549 cells in 50% Matrigel (BD Biosciences, Franklin Lakes, NJ, USA) were injected on the flank of Balb/C nude mice, which were randomly separated into two groups for treatment of 2 mg/kg scrambled siRNA and USP47 siRNA (4 mice per group). When the tumors reached ~100 mm^3^, treatments were conducted. Scrambled siRNA and USP47 siRNA were locally injected every 3 days for 2 weeks using lipofectamine 3000 kit (Life Technologies, Carlsbad, CA, USA) according to the manufacturer’s instruction. Tumors were monitored, and tumor volumes were measured every 3 days by a digital caliper. Tumor volumes were determined by the formula V = (length × width^2^)/2. Xenografts were sacrificed at 33 days post initial treatment, and subcutaneous tumors were dissected for immunofluorescence and apoptosis studies. Statistical significance between treatment and control groups was evaluated by one-way ANOVA followed by Tukey’s test.

### 4.11. Tissue Immunofluorescence Staining and TUNEL Assay

Immunofluorescence analyses were conducted on frozen sections of dissected tumors. After death, tumors were embedded in OCT (Leica, Wetzlar, Germany), and blocks were sectioned (7 µm thickness) using a cryostat (CM1900, Leica). Frozen sections were accomplished by a standard immunostaining procedure. Briefly, sections were incubated with primary anti-USP47 (Bethyl, 1:333 dilution), anti-p53 (Santa Cruz, 1:333 dilution), and anti-MDM2 (Calbiochem, 1:333 dilution) antibodies at 4 °C overnight, respectively, followed by Alexa Fluor-488-labeled anti-rabbit (Life Technologies). Tissues were counterstained with mounting medium (Vector Laboratories, Burlingame, CA, USA) containing DAPI solution. For the detection of apoptotic cells, the tumor sections were stained using an in situ cell death detection kit (Roche) according to the manufacturer’s instruction. Sections were counterstained with mounting medium. All fluorescence images were obtained by confocal microscopy (LSM700, Carl Zeiss, Oberkochen, Germany) and processed by ZEN software (Carl Zeiss). Fluorescence intensities were quantified by ImageJ software (NIH, Bethesda, MD, USA).

### 4.12. Immunofluorescence

We followed the immunofluorescence protocol of Abcam. Sample coverslips were fixed in ice-cold methanol for 10 min at room temperature and washed three times with cold PBS for 5 min. Samples were incubated with 1% BSA in TBST for 30 min to block nonspecific binding of antibodies. After blocking, samples were incubated with anti-HA (Santa Cruz) or anti-Flag (Sigma Aldrich) antibodies followed by secondary goat anti-rabbit antibody coupled to Alexa Fluor 488 (Invitrogen) and goat anti-mouse antibody coupled to Alexa Fluor 546 (Invitrogen). The samples were then incubated with 2 μg/mL DAPI for 10 min and washed with PBS. Coverslips were mounted with a drop of mounting medium (Dako, #S3023, Glostrup, Denmark). Fluorescence was visualized with X630 magnification on a confocal laser scanning microscope (Carl Zeiss, Inc. LSM700), and the images were analyzed with ZEN 2009 software.

## 5. Conclusions

This study illustrated that USP47 functions as an important regulator of p53. We found RPS2 is a substrate of USP47 DUB activity, and USP47 counteracts MDM2 to RPS2 ubiquitination, which in turn can regulate p53. Through the deubiquitination of RPS2, USP47 regulates the interaction between RPS2 and MDM2, and USP47 functions as an important regulator of the MDM2–p53 axis in the ribosomal stress. Finally, we provide evidence that USP47 has oncogenic potential, and depletion of USP47 induces p53 by regulation of RPS2 and, therefore, inhibits cell proliferation, colony formation, and tumor progression in cancer cell lines and a mouse xenograft model in a p53-dependent manner. Therefore, USP47 could well be a potential therapeutic target for cancer.

## Supplementary Materials

The following are available online at https://www.mdpi.com/2072-6694/12/5/1137/s1, Figure S1: Overexpressed USP47 interacts with endogenous RPS2 in cells. Figure S2: USP47 depletion induces p53 protein level. Figure S3: MDM2 does not have any effect on the interaction between USP47 and RPS2. Figure S4: MDM2 ubiquitination is increased by RPS2 and restored by USP47. Figure S5: The ubiquitination of endogenous RPS2 is accumulated with Actinomycin D treatment in a time-dependent manner. Figure S6: Inhibition of xenograft tumor by knockdown of USP47. Figure S7: The uncropped blots and molecular weight markers of the Figure 1 Figure 2 Figure 3 Figure 4 Figure 5 Figure 6 Figure 7 in main text.

## References

[1] Liu D., Xu Y. (2011). P53, oxidative stress, and aging. Antioxid. Redox Signal..

[2] Vousden K.H., Prives C. (2009). Blinded by the light: The growing complexity of p53. Cell.

[3] Kruse J.P., Gu W. (2009). Modes of p53 regulation. Cell.

[4] Brooks C.L., Gu W. (2006). P53 ubiquitination: Mdm2 and beyond. Mol. Cell.

[5] Li M., Chen D., Shiloh A., Luo J., Nikolaev A.Y., Qin J., Gu W. (2002). Deubiquitination of p53 by hausp is an important pathway for p53 stabilization. Nature.

[6] Khoronenkova S.V., Dianova I.I., Ternette N., Kessler B.M., Parsons J.L., Dianov G.L. (2012). Atm-dependent downregulation of usp7/hausp by ppm1g activates p53 response to DNA damage. Mol. Cell.

[7] Yuan J., Luo K., Zhang L., Cheville J.C., Lou Z. (2010). Usp10 regulates p53 localization and stability by deubiquitinating p53. Cell.

[8] Zhang L., Nemzow L., Chen H., Lubin A., Rong X., Sun Z., Harris T.K., Gong F. (2015). The deubiquitinating enzyme usp24 is a regulator of the uv damage response. Cell Rep..

[9] Hock A.K., Vigneron A.M., Carter S., Ludwig R.L., Vousden K.H. (2011). Regulation of p53 stability and function by the deubiquitinating enzyme usp42. EMBO J..

[10] Stevenson L.F., Sparks A., Allende-Vega N., Xirodimas D.P., Lane D.P., Saville M.K. (2007). The deubiquitinating enzyme usp2a regulates the p53 pathway by targeting mdm2. EMBO J..

[11] Palazon-Riquelme P., Worboys J.D., Green J., Valera A., Martin-Sanchez F., Pellegrini C., Brough D., Lopez-Castejon G. (2018). Usp7 and usp47 deubiquitinases regulate nlrp3 inflammasome activation. EMBO Rep..

[12] Zhang X., Berger F.G., Yang J., Lu X. (2011). Usp4 inhibits p53 through deubiquitinating and stabilizing arf-bp1. EMBO J..

[13] Lin Z., Yang H., Kong Q., Li J., Lee S.M., Gao B., Dong H., Wei J., Song J., Zhang D.D. (2012). Usp22 antagonizes p53 transcriptional activation by deubiquitinating sirt1 to suppress cell apoptosis and is required for mouse embryonic development. Mol. Cell.

[14] Sun X.X., Challagundla K.B., Dai M.S. (2012). Positive regulation of p53 stability and activity by the deubiquitinating enzyme otubain 1. EMBO J..

[15] Zhou X., Liao W.J., Liao J.M., Liao P., Lu H. (2015). Ribosomal proteins: Functions beyond the ribosome. J. Mol. Cell Biol..

[16] Bursac S., Brdovcak M.C., Pfannkuchen M., Orsolic I., Golomb L., Zhu Y., Katz C., Daftuar L., Grabusic K., Vukelic I. (2012). Mutual protection of ribosomal proteins l5 and l11 from degradation is essential for p53 activation upon ribosomal biogenesis stress. Proc. Natl. Acad. Sci. USA.

[17] Zhu Y., Poyurovsky M.V., Li Y., Biderman L., Stahl J., Jacq X., Prives C. (2009). Ribosomal protein s7 is both a regulator and a substrate of mdm2. Mol. Cell.

[18] Sun X.X., DeVine T., Challagundla K.B., Dai M.S. (2011). Interplay between ribosomal protein s27a and mdm2 protein in p53 activation in response to ribosomal stress. J. Biol. Chem..

[19] Cho J., Park J., Shin S.C., Kim J.H., Kim E.E., Song E.J. (2020). Ribosomal protein s2 interplays with mdm2 to induce p53. Biochem. Biophys. Res. Commun..

[20] Turnbull A.P., Ioannidis S., Krajewski W.W., Pinto-Fernandez A., Heride C., Martin A.C.L., Tonkin L.M., Townsend E.C., Buker S.M., Lancia D.R. (2017). Molecular basis of usp7 inhibition by selective small-molecule inhibitors. Nature.

[21] Kategaya L., Di Lello P., Rouge L., Pastor R., Clark K.R., Drummond J., Kleinheinz T., Lin E., Upton J.P., Prakash S. (2017). Usp7 small-molecule inhibitors interfere with ubiquitin binding. Nature.

[22] Lamberto I., Liu X., Seo H.S., Schauer N.J., Iacob R.E., Hu W., Das D., Mikhailova T., Weisberg E.L., Engen J.R. (2017). Structure-guided development of a potent and selective non-covalent active-site inhibitor of usp7. Cell Chem. Biol..

[23] Poondla N., Chandrasekaran A.P., Kim K.S., Ramakrishna S. (2019). Deubiquitinating enzymes as cancer biomarkers: New therapeutic opportunities?. BMB Rep..

[24] Faesen A.C., Luna-Vargas M.P., Sixma T.K. (2012). The role of ubl domains in ubiquitin-specific proteases. Biochem. Soc. Trans..

[25] Kayama K., Watanabe S., Takafuji T., Tsuji T., Hironaka K., Matsumoto M., Nakayama K.I., Enari M., Kohno T., Shiraishi K. (2017). Grwd1 negatively regulates p53 via the rpl11-mdm2 pathway and promotes tumorigenesis. EMBO Rep..

[26] Sasaki M., Kawahara K., Nishio M., Mimori K., Kogo R., Hamada K., Itoh B., Wang J., Komatsu Y., Yang Y.R. (2011). Regulation of the mdm2-p53 pathway and tumor growth by pict1 via nucleolar rpl11. Nat. Med..

[27] Fang Z., Cao B., Liao J.M., Deng J., Plummer K.D., Liao P., Liu T., Zhang W., Zhang K., Li L. (2018). Spin1 promotes tumorigenesis by blocking the ul18 (universal large ribosomal subunit protein 18)-mdm2-p53 pathway in human cancer. eLife.

[28] Holmberg Olausson K., Nister M., Lindstrom M.S. (2012). P53 -dependent and -independent nucleolar stress responses. Cells.

[29] Ashcroft M., Taya Y., Vousden K.H. (2000). Stress signals utilize multiple pathways to stabilize p53. Mol. Cell. Biol..

[30] Choi B.J., Park S.A., Lee S.Y., Cha Y.N., Surh Y.J. (2017). Hypoxia induces epithelial-mesenchymal transition in colorectal cancer cells through ubiquitin-specific protease 47-mediated stabilization of snail: A potential role of sox9. Sci. Rep..

[31] Parsons J.L., Dianova I.I., Khoronenkova S.V., Edelmann M.J., Kessler B.M., Dianov G.L. (2011). Usp47 is a deubiquitylating enzyme that regulates base excision repair by controlling steady-state levels of DNA polymerase beta. Mol. Cell.

[32] Peschiaroli A., Skaar J.R., Pagano M., Melino G. (2010). The ubiquitin-specific protease usp47 is a novel beta-trcp interactor regulating cell survival. Oncogene.

[33] Macias E., Jin A., Deisenroth C., Bhat K., Mao H., Lindstrom M.S., Zhang Y. (2010). An arf-independent c-myc-activated tumor suppression pathway mediated by ribosomal protein-mdm2 interaction. Cancer Cell.

[34] Mahata B., Sundqvist A., Xirodimas D.P. (2012). Recruitment of rpl11 at promoter sites of p53-regulated genes upon nucleolar stress through nedd8 and in an mdm2-dependent manner. Oncogene.

[35] Ofir-Rosenfeld Y., Boggs K., Michael D., Kastan M.B., Oren M. (2008). Mdm2 regulates p53 mrna translation through inhibitory interactions with ribosomal protein l26. Mol. Cell.

[36] Donati G., Peddigari S., Mercer C.A., Thomas G. (2013). 5s ribosomal rna is an essential component of a nascent ribosomal precursor complex that regulates the hdm2-p53 checkpoint. Cell Rep..

[37] Sloan K.E., Bohnsack M.T., Watkins N.J. (2013). The 5s rnp couples p53 homeostasis to ribosome biogenesis and nucleolar stress. Cell Rep..

[38] Zhou J., Wang J., Chen C., Yuan H., Wen X., Sun H. (2018). Usp7: Target validation and drug discovery for cancer therapy. Med. Chem..

[39] Fan Y.H., Cheng J., Vasudevan S.A., Dou J., Zhang H., Patel R.H., Ma I.T., Rojas Y., Zhao Y., Yu Y. (2013). Usp7 inhibitor p22077 inhibits neuroblastoma growth via inducing p53-mediated apoptosis. Cell Death Dis..

[40] Wang M., Zhang Y., Wang T., Zhang J., Zhou Z., Sun Y., Wang S., Shi Y., Luan X., Zhang Y. (2017). The usp7 inhibitor p5091 induces cell death in ovarian cancers with different p53 status. Cell. Physiol. Biochem..

[41] Weinstock J., Wu J., Cao P., Kingsbury W.D., McDermott J.L., Kodrasov M.P., McKelvey D.M., Suresh Kumar K.G., Goldenberg S.J., Mattern M.R. (2012). Selective dual inhibitors of the cancer-related deubiquitylating proteases usp7 and usp47. ACS Med. Chem. Lett..

[42] Barboza J.A., Iwakuma T., Terzian T., El-Naggar A.K., Lozano G. (2008). Mdm2 and mdm4 loss regulates distinct p53 activities. Mol. Cancer Res..

[43] Kingston R.E., Chen C.A., Okayama H. (2003). Calcium phosphate transfection. Curr. Protoc. Cell Biol..

[44] Yang S.W., Oh K.H., Park E., Chang H.M., Park J.M., Seong M.W., Ka S.H., Song W.K., Park D.E., Baas P.W. (2013). Usp47 and c terminus of hsp70-interacting protein (chip) antagonistically regulate katanin-p60-mediated axonal growth. J. Neurosci..

[45] Kim H., Lee J.M., Lee G., Bhin J., Oh S.K., Kim K., Pyo K.E., Lee J.S., Yim H.Y., Kim K.I. (2011). DNA damage-induced roralpha is crucial for p53 stabilization and increased apoptosis. Mol. Cell.

[46] Song E.J., Werner S.L., Neubauer J., Stegmeier F., Aspden J., Rio D., Harper J.W., Elledge S.J., Kirschner M.W., Rape M. (2010). The prp19 complex and the usp4sart3 deubiquitinating enzyme control reversible ubiquitination at the spliceosome. Genes Dev..

[47] Park J., Kwon M.S., Kim E.E., Lee H., Song E.J. (2018). Usp35 regulates mitotic progression by modulating the stability of aurora b. Nat. Commun..

[48] Song H., Li D., Wu T., Xie D., Hua K., Hu J., Deng X., Ji C., Deng Y., Fang L. (2018). Microrna-301b promotes cell proliferation and apoptosis resistance in triple-negative breast cancer by targeting cyld. BMB Rep..

