# Supplementary material for: USP47 Promotes Tumorigenesis by Negative Regulation of p53 through Deubiquitinating Ribosomal Protein S2
Source: Cancers (Basel). 2020 May 1;12(5):1137. doi: 10.3390/cancers12051137 (PMC7281321; doi:10.3390/cancers12051137)

cancers

MDPI

Article

Supplementary Materials: USP47 Promotes Tumorigenesis by Negative Regulation of p53 through Deubiquitinating Ribosomal Protein S2

Jinhong Cho, Jinyoung Park, Sang Chul Shin, Mihue Jang, Jae-Hong Kim, Eunice EunKyeong Kim and Eun Joo Song

Supplementary Materials

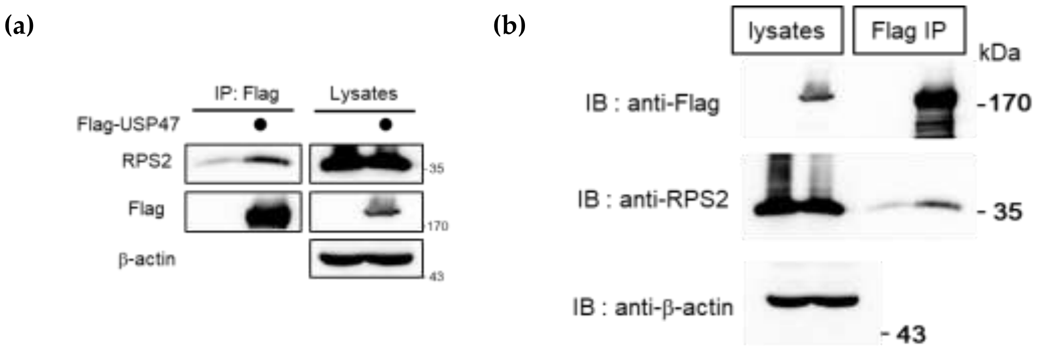

Figure S1. Overexpressed USP47 interacts with endogenous RPS2 in cells. (**a**) HEK293T cells were overexpressed with Flag vector or Flag-USP47, and the cell lysates were then immunoprecipitated with anti-Flag M2 agarose and detected with the indicated antibodies, and β-actin was used as a loading control. (**b**) Uncropped Western Blot figure.

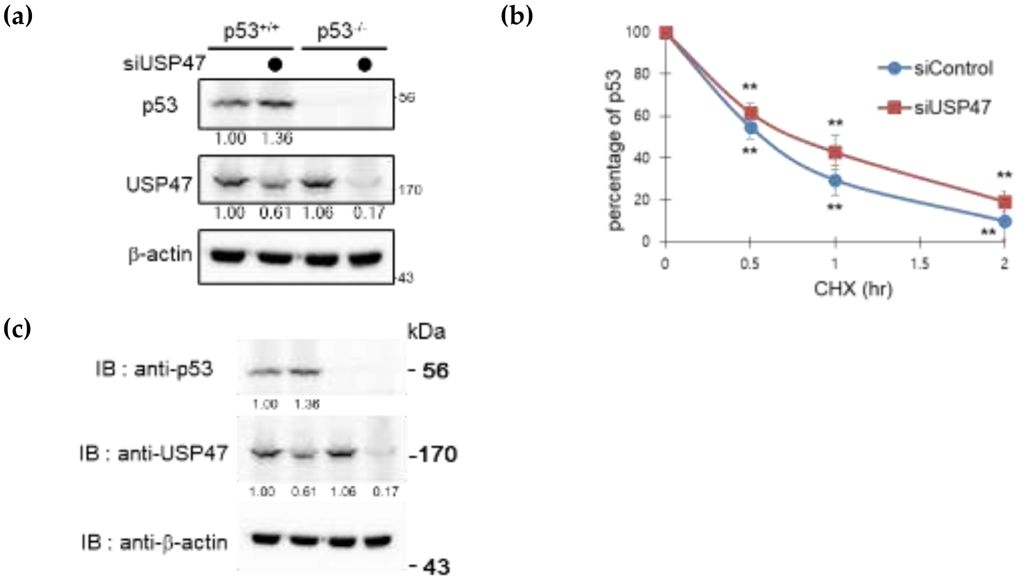

Figure S2. USP47 depletion induces p53 protein level. (**a**) HCT116 p53$^{+/+}$ and p53$^{-/-}$ cells were transfected with siRNA against control or USP47 for 72 h. Cell lysates were immunoblotted with the indicated antibodies. (**b**) U2OS cells transfected with siRNA against control or USP47 were treated with 100 µg/ml cycloheximide and harvested at the indicated times. Cell lysates were immunoblotted with the indicated antibodies. The band intensity was measured by Image J and then p53 levels are normalized by HSP90. The data from three independent experiments represent the mean ± SD (* $p < 0.05$, ** $p < 0.01$, *t*-test). (**c**) Uncropped Western Blot figure.

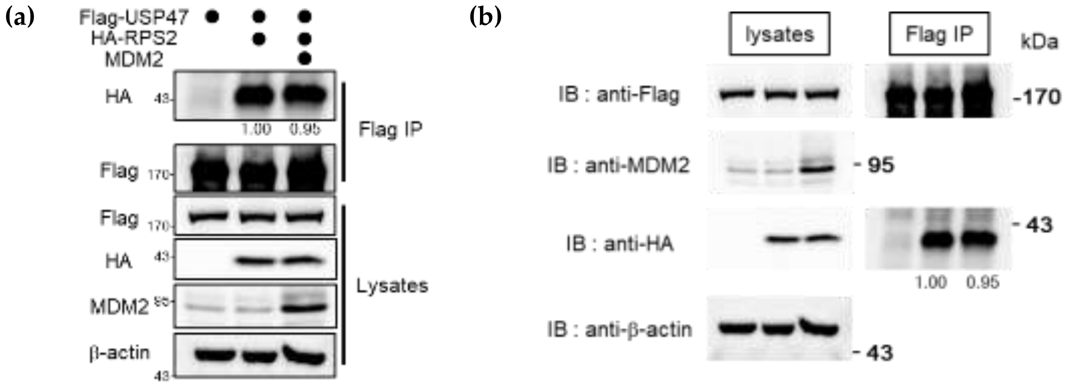

Figure S3. (**a**)MDM2 does not have any effect on the interaction between USP47 and RPS2. HEK293T cells were co-overexpressed with Flag-USP47, HA-RPS2, MDM2 and then, immunoprecipitation was performed with anti-Flag M2 agarose. Bound proteins were immunoblotted with the indicated antibodies. (**b**) Uncropped Western Blot figure.

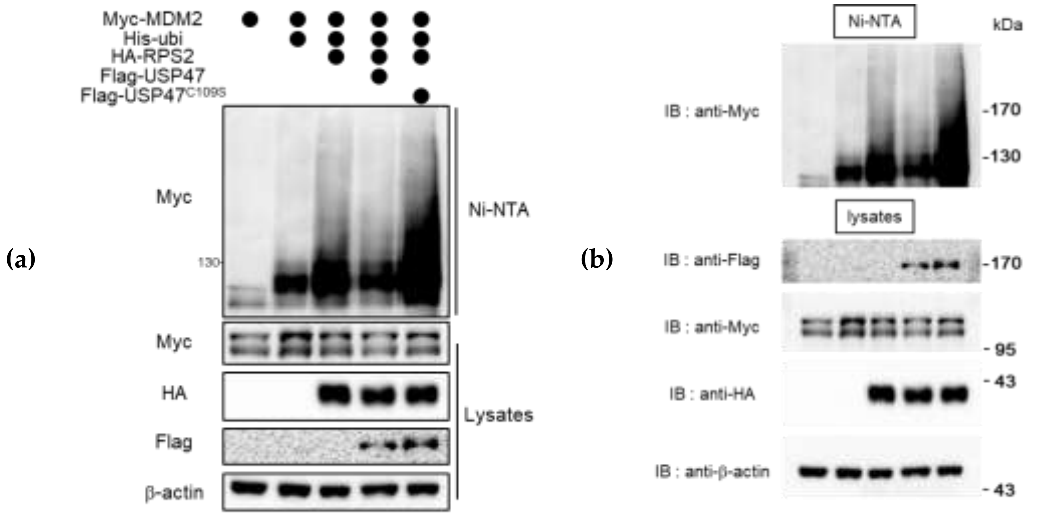

Figure S4. (**a**) MDM2 ubiquitination is increased by RPS2 and restored by USP47. HEK293T cells were co-overexpressed with Myc-MDM2, His-ubiquitin, HA-RPS2, Flag-USP47, or Flag-USP47C109S for 24 h as indicated and then treated with 10 μM MG132 for 4 h. Ubiquitin conjugates were purified on Ni-NTA-agarose under denaturing conditions, and ubiquitinated MDM2 was detected by anti-Myc antibodies. (**b**) Uncropped Western Blot figure.

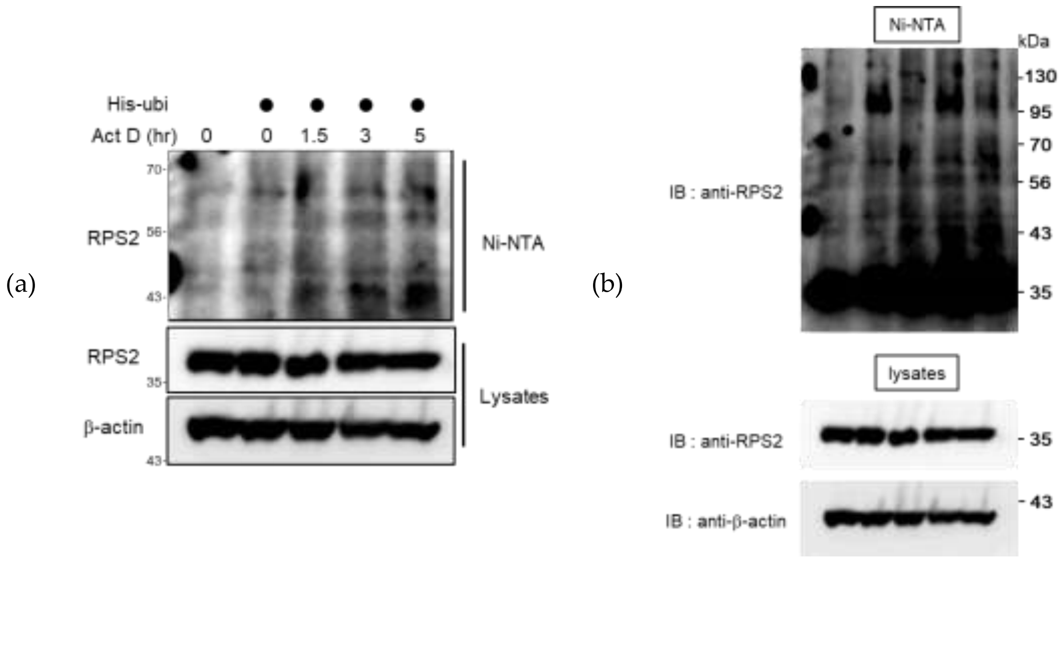

Figure S5. (a) The ubiquitination of endogenous RPS2 is accumulated with Actinomycin D treatment in a time-dependent manner. HEK293T cells were overexpressed with His-ubiquitin for 24 h and then treated with 10 μM MG132 for 4 h and 5 nM actinomycin D for indicated times. Ubiquitin conjugates were purified on Ni-NTA-agarose under denaturing conditions, and ubiquitinated endogenous RPS2 was detected by anti-RPS2 antibodies. (**b**) Uncropped Western Blot figure.

(a)

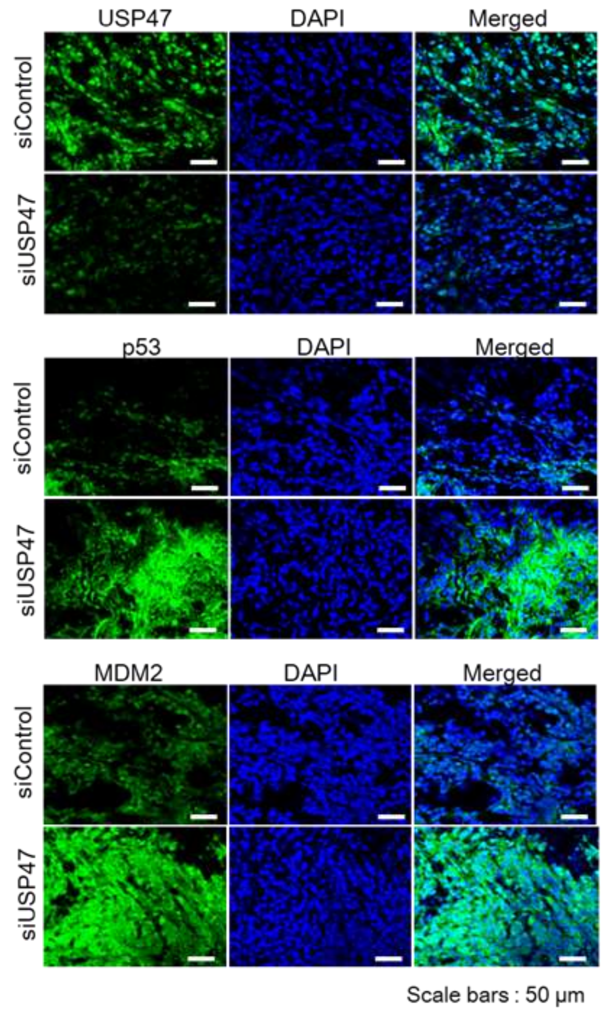

(b)

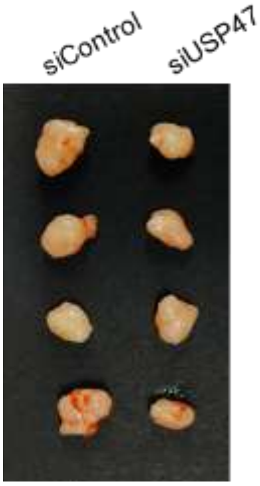

Figure S6. Inhibition of xenograft tumor by knockdown of USP47. (**a**,**b**) 2 mg/kg of siControl and siUSP47 were intratumorally administered into A549 xenografts every 3 days for 2 weeks. (**a**) Immunofluorescence staining was conducted in dissected tumors and detected by indicated antibodies. Immunofluorescence images were captured by confocal microscopy. Blue colors represent DAPI. Scale bars indicate 50 μm. (**b**) Xenograft tumor images.

Figure S7. The uncropped **Western Blots** and molecular weight markers of the Figures 1–7 in main text.

Figure 1

(a)

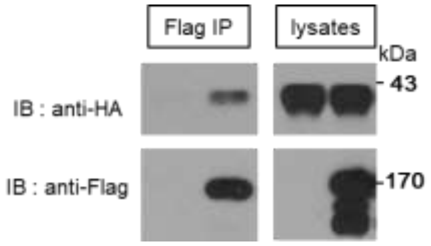

(b)

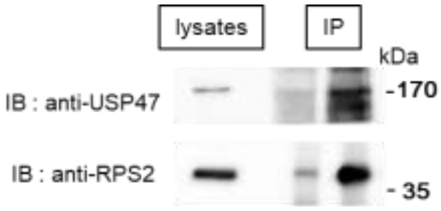

(c)

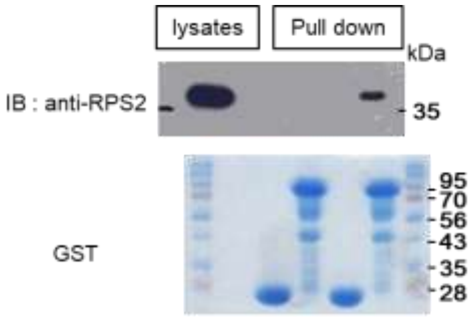

(d)

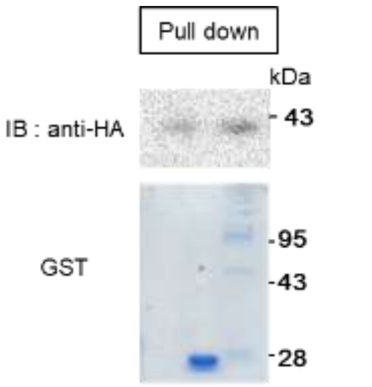

Figure 2

(a)

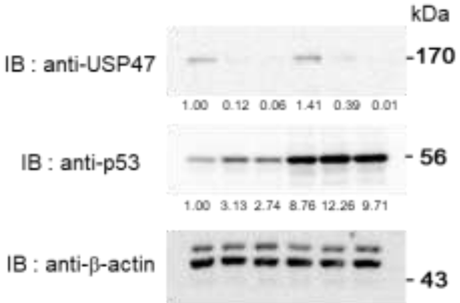

(b)

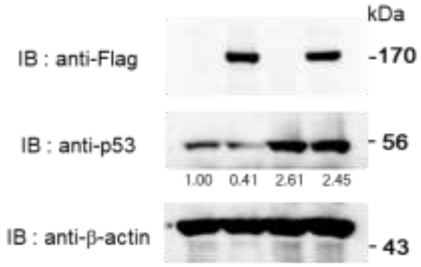

(c)

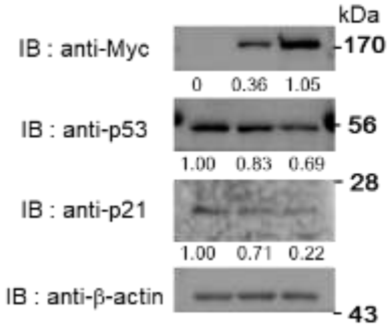

(d)

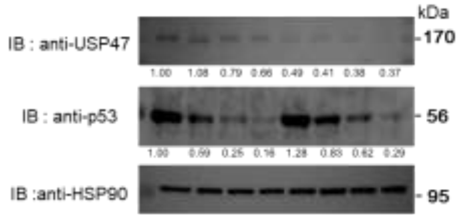

(e)

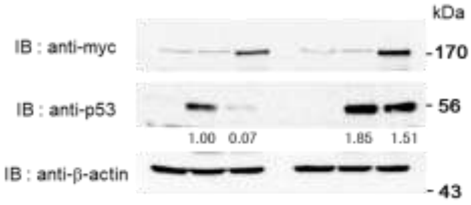

(f)

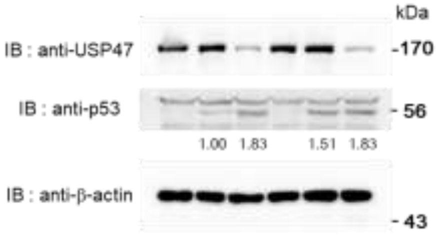

Figure 3

(a)

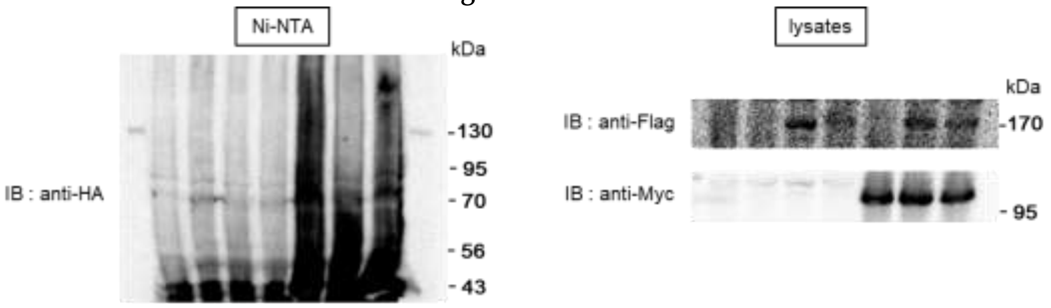

Figure 3

(b)

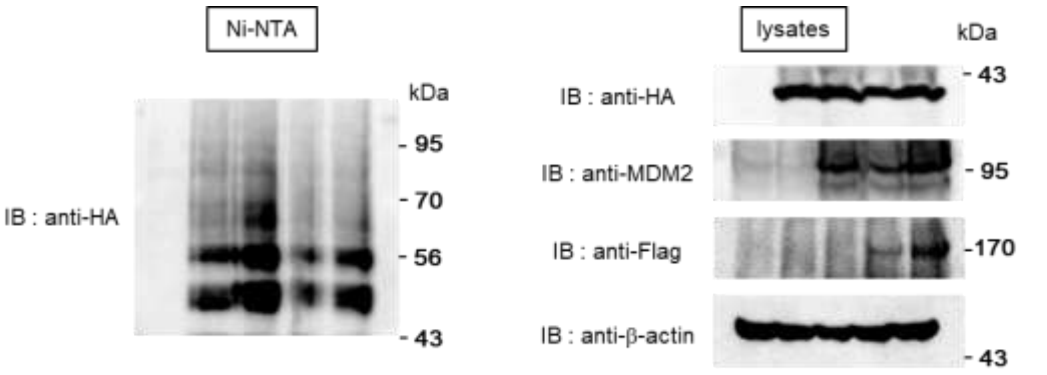

(c)

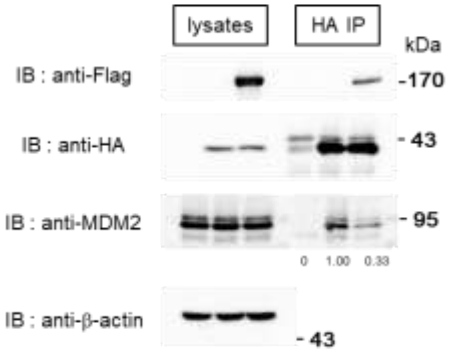

(d)

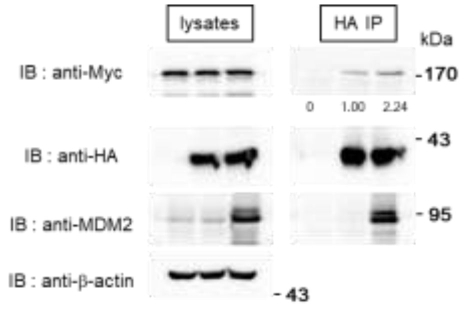

Figure 4

(a)

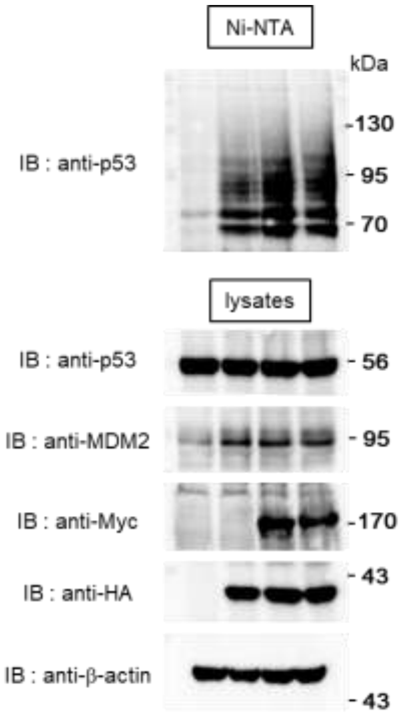

(b)

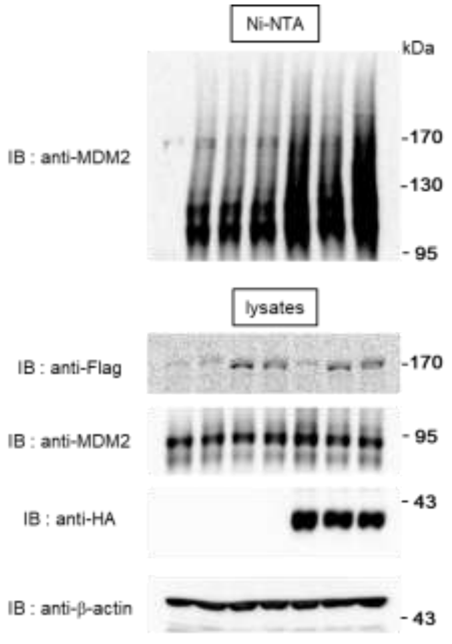

(c)

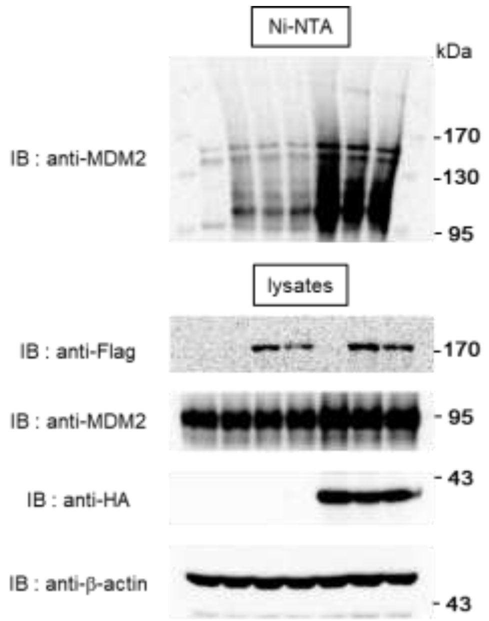

(d)

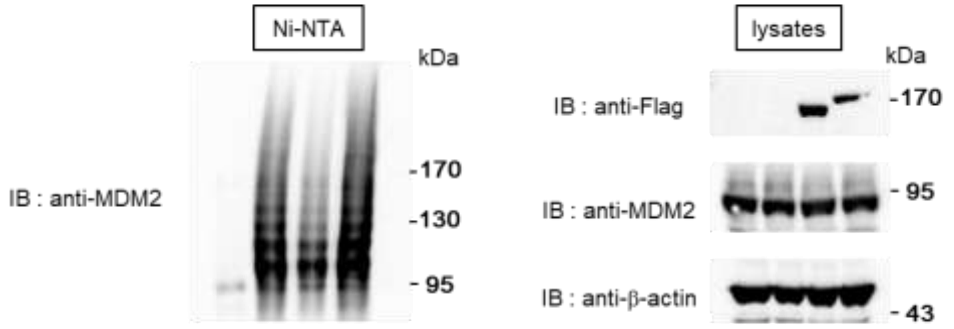

Figure 5

(a)

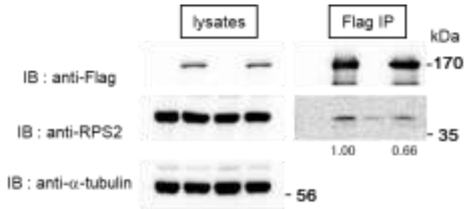

(b)

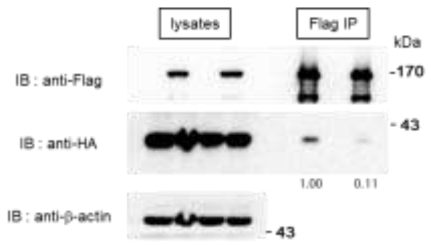

(d)

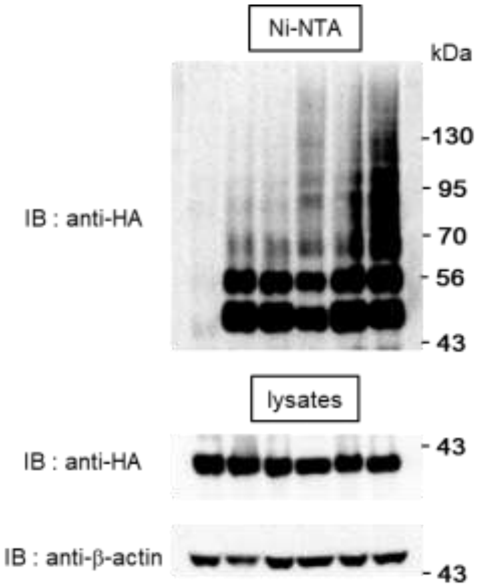

(e)

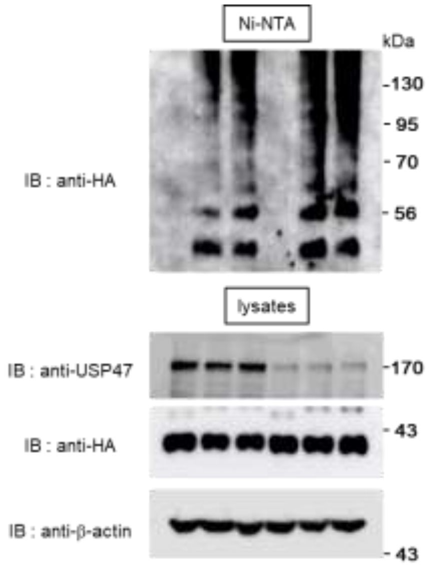

(f)

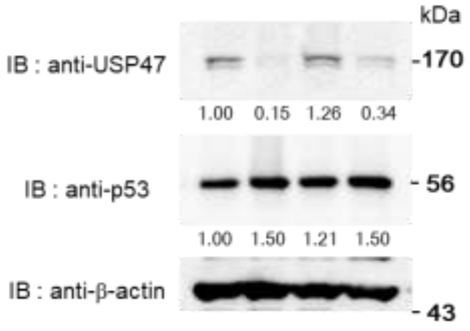

Figure 6

(a)

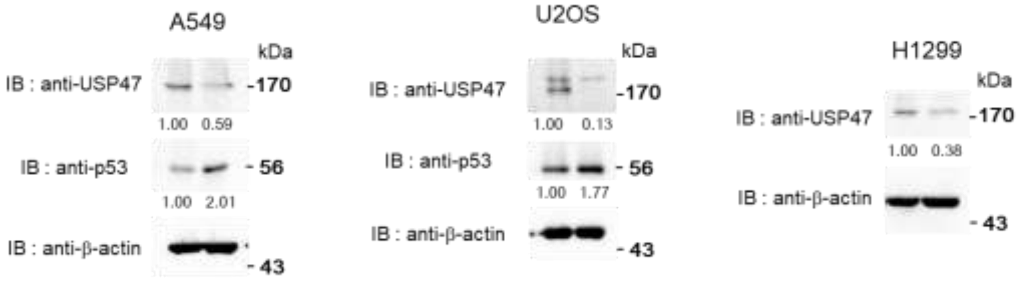

Figure 7

(e)

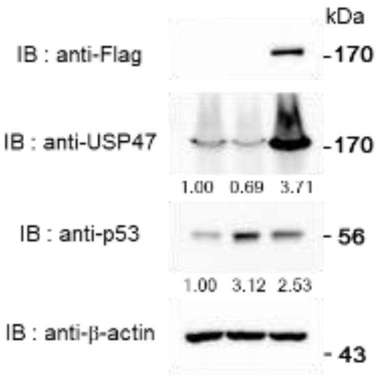

(a)

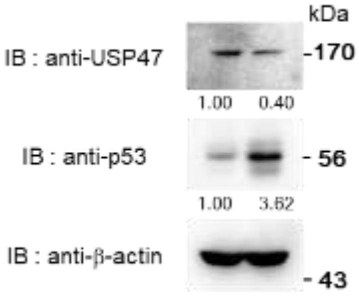

Supplement: Supplementary file 1 [file cancers-12-01137-s001.pdf]
